# Editorial for the Special Issue “Gels for Removal and Adsorption (2nd Edition)”

**DOI:** 10.3390/gels10080512

**Published:** 2024-08-03

**Authors:** Zhenxing Fang, Kaiming Peng, Shiyang Li

**Affiliations:** 1 Ningbo Key Laboratory of Agricultral Germplasm Resources Mining and Environmental Regulation, College of Science and Technology, Ningbo University, Ningbo 315300, China; fangzhenxing128@163.com; 2 College of Environmental Science and Engineering, Institution of Carbon Neutrality, Tongji University, Shanghai 200092, China; pengkaiming@tongji.edu.cn; 3 Key Laboratory of Organic Compound Pollution Control Engineering (MOE), School of Environmental and Chemical Engineering, Shanghai University, Shanghai 200444, China

## 1. Introduction

Gel materials, especially hydrogels and aerogels, have been materials of interest in adsorption technology research in recent years [1]. Hydrogels are crosslinked networks of hydrophilic polymers, which contain a large amount of water without dissolving, maintaining their three-dimensional network structure [2]. This structure endows hydrogels with excellent adsorption performance, enabling them to remove and adsorb water-soluble pollutant molecules. Aerogel is a type of material with high porosity and low density. Its unique three-dimensional network structure grants it excellent performance in the field of gas adsorption and purification [3].

The applications of hydrogel in wastewater treatment have made remarkable progress. Due to the rich groups, porous structure, and convenient controllability, hydrogels have significant advantages in the adsorption and purification of wastewater. Harmful substances such as heavy metal ions, organic pollutants, and dyes from wastewater could be efficiently removed by the functional hydrogels [4,5,6]. However, the hydrogel adsorbents prepared at present still face problems, such as insufficient binding groups and poor mechanical properties, which limit their practical application. Therefore, the design and preparation of novel hydrogel adsorbents with good adsorption properties and mechanical properties is a current research hotspot. The performance of hydrogels could be improved by introducing functional groups, adjusting the degree of crosslinking, and combining them with other materials [7,8]. For example, the introduction of amino, carboxyl, and other functional groups can enhance the selective adsorption capacity of hydrogels for specific pollutants. The adsorption property and mechanical strength of the hydrogel can be balanced by adjusting the crosslinking degree. The adsorption efficiency and stability of hydrogels can be further improved by combining them with other materials such as nanoparticles, fibers, etc.

The applications of aerogels in the field of gas adsorption purification are also remarkable. Inorganic oxide aerogels, such as SiO_2_ aerogels, have attracted much attention due to their high adsorption efficiency, convenient desorption, and stable performance [9,10]. After amino functionalization, the selective adsorption capacity for CO_2_ was significantly improved, maintaining stable performance in multiple adsorption and desorption cycles. In addition, new aerogel materials such as metal oxide aerogel and carbon aerogel gel also show unique advantages in gas adsorption and purification.

Gel materials have a broad application prospect in the field of adsorption and removal. With the continuous progress of science and technology and in-depth research, the development and application of new gel materials will continue to promote the development of this field. In the future, we expect to see more high-performance and multi-functional gel materials widely used in wastewater treatment, gas purification, and other fields, providing strong support for solving environmental pollution problems. In this context, this Special Issue, entitled “Gels for Removal and Adsorption (2nd Edition)”, in *Gels*, has been established to shed light on gels’ synthesis with various biomasses, inorganic and organic materials, crosslinking, structural characterization, and the applications for removal and adsorption. In addition to this Editorial, this Special Issue is comprised of 10 articles in total. It is encouraging that these contributions can boost the development of gels’ applications, especially in the field of pollutant removal and adsorption. The following content will provide a brief overview of them.

## 2. Overview

### 2.1. Some Novel Methods to Synthesize Functional Hydrogels

Zhou et al. reported that a novel lignocellulose-based aerogel was fabricated by partially dissolving medulla tetrapanacis via ionic liquid. The prepared aerogels preserve the original honeycomb-like porous structure, and the newly formed micropores due to the partial dissolving by ionic liquid [11]. The formed micropores enhance the material’s capillary force, providing efficient directional transport performance, making it well suited for applications requiring high compressive performance and selective directional transport.

Wang et al. reported that a water-driven recovery aerogel was prepared by crosslinking epoxy groups with chitosan amino groups. The main structure of epoxy groups is oligomeric silsesquioxane, which enhances its susceptibility to deformation [12]. The synthesized aerogels exhibit excellent water-driven recovery performance, regaining their original volume within a very short time (1.9 s) after strong compression (ε > 80%).

Wang et al. reported that N-(3-Dimethylaminopropyl)-N’-ethylcarbodiimide hydrochloride (EDC) and N-Hydroxysuccinimide (NHS) was used to crosslink silicon-doped carbon dots and carboxymethyl cellulose. The silicon-doped carbon dots crosslinked gels show improved mechanical properties but also good biocompatibility, reactivity, and fluorescence properties [13]. The abundant crosslinking points endow the gel with excellent mechanical properties, with a compressive strength reaching 294 kPa.

### 2.2. Applications for Wastewater Pollutant Removal and Adsorption

Hydrogels are widely used for wastewater treatment. In this Special Issue, four articles are closely related to wastewater treatment. A brief introduction is listed as follows:

An article from Argentina, entitled “Linear Polyethyleneimine-Based and Metal Organic Frameworks (DUT-67) Composite Hydrogels as Efficient Sorbents for the Removal of Methyl Orange, Copper Ions, and Penicillin V”, introduced that highly efficient composite hydrogen adsorbents could be fabricated by integrating DUT-67 metal organic frameworks into polyethyleneimine-based hydrogels. The particle size of DUT-67 was successfully modulated from 1 µm to 200 nm by varying the solvent-to-modulator ratio in a water-based synthesis [14]. The obtained hydrogel with enough mechanical strength, pore structure, and chemical affinity exhibits maximum adsorption capacities of 473 ± 21 mg L^−1^, 86 ± 6 mg L^−1^, and 127 ± 4 mg L^−1^, for methyl orange, copper(II) ions, and penicillin V, respectively. This hydrogel reveals a promising application as an efficient adsorbent for environmental pollutants and pharmaceuticals.

An article from Italy, entitled “Preparation of Peptide-Based Magnetogels for Removing Organic Dyes from Water”, reported that polyacrylic acid-modified γ-Fe_2_O_3_ nanoparticles were embedded in hydrogel through the enzymatic approach. The ability of the magnetogel to remove three organic dyes, methyl orange, methylene blue, and rhodamine 6G, was studied. The results show that the obtained peptide magnetogel could represent a valuable and environmentally friendly alternative to currently employed adsorbents [15].

An article from China, entitled “Study on Adsorption of Cd in Solution and Soil by Modified Biochar–Calcium Alginate Hydrogel”, reported a fabricating composite material method by utilizing crosslinked modified biochar (prepared from pine wood) and calcium alginate hydrogels. The modified biochar and calcium alginate hydrogels exhibit a higher heavy metal adsorption capacity compared to traditional biochar and hydrogels due to their increased oxygen-containing functional groups and heavy metal adsorption sites [16]. The highest Cd^2+^ removal rate of the obtained hydrogel reached up to 85.48%. This study gives a novel approach for managing Cd-contaminated cultivated land.

Another article from China, entitled “Molten Alkali-Assisted Formation of Silicate Gels and Its Application for Preparing Zeolites”, illustrated the convenient formation of silicate gels assisted by molten alkali, which was energy and time saving [17]. The silicate gels were used to fabricate zeolite successfully. The maximum adsorption capacity of obtained zeolite for ammonium can reach 49.1 mg/g. The ammonium-adsorbed zeolites might be used as an environmentally friendly ammonium fertilizer for agricultural plant growth.

### 2.3. Some Other Applications for Arts Remediation and Medical Care

Carretti et al. reported that a non-aqueous organogel sponge made of PDMS was used for the cleaning of artworks. The role of pore size in an elastomeric network on the ability to uptake and release organic material was studied in detail. Two different sugar templates were applied to synthesize porous organic polymers, whose porosity drops with the decrease in pore size [18]. The adsorption capacity was measured by swelling with eight solvents covering a wide range of polarities. The results demonstrate that the PDMS sponges are a potential innovative support for the controlled and selective cleaning of art surfaces.

Barati et al. reported that a chitosan-based low-cost microneedle patch was obtained by a CO_2_ laser cutter [19]. The impact of Glycyrrhiza glabra extract (GgE), delivered via microneedle to the cell population on the patch, was evaluated. A lot of analysis, such as microscopic analysis, swelling, penetration, degradation, biocompatibility, and drug delivery were carried out to assess the gel-based microneedle patch’s performance. In general, a GgE-loaded microneedle patch can be a good remedy for skin disorders in which cell proliferation needs to be controlled.

Gabriele reported that a series of chitosan-carboxylic acid hydrogels was prepared by coupling the reducing ability of carboxylic acids with the intrinsic chelating properties of the polysaccharide [20]. The results show that the formulation containing oxalic acid is the most effective in removing rust stains. This work developed an effective formulation for removing rust on a marble surface, which is the most challenging surface to clean.

## 3. Summary

In summary, this Special Issue involved a number of interesting research articles that have represented the most recent progress in various aspects of gels, including the preparation technique, the applications for pollutant removal and adsorption, the treatment of artworks, and medical care. These gels may offer new insights into the design, preparation, development, and application of biomass-based, inorganic, and organic gels. We greatly appreciate the efforts of our authors, reviewers, and editors in the disclosure of these valuable research works.
